# Heterogeneous Landscapes on Steep Slopes at Low Altitudes as Hotspots of Bird Diversity in a Hilly Region of Nepal in the Central Himalayas

**DOI:** 10.1371/journal.pone.0150498

**Published:** 2016-03-03

**Authors:** Tej B. Basnet, Maan B. Rokaya, Bishnu P. Bhattarai, Zuzana Münzbergová

**Affiliations:** 1 Central Department of Zoology, Tribhuvan University, Kirtipur, Nepal; 2 Institute of Botany, Czech Academy of Sciences, Zamek 1, CZ-252 43, Průhonice, Czech Republic; 3 Department of Biodiversity Research, Global Change Research Institute, Czech Academy of Sciences, Bělidla 4a, 603 00, Brno, Czech Republic; 4 Birendra Multiple Campus, Tribuvan University, Kirtipur, Nepal; 5 Department of Botany, Faculty of Science, Charles University, Benatska 2, 128 01, Prague, Czech Republic; Estación Biológica de Doñana, CSIC, SPAIN

## Abstract

Understanding factors determining the distribution of species is a key requirement for protecting diversity in a specific area. The aim of this study was to explore the factors affecting diversity and distribution of species of birds on different forested hills in central Nepal. The area is rich in species of birds. Because the area is characterized by steep gradients, we were also interested in the importance of altitude in determining the diversity and species composition of the bird communities. We assessed bird diversity and species composition based on point observations along a gradient of increasing altitude in two valleys (Kathmandu and Palung) in central Nepal. Data on environmental variables were also collected in order to identify the main determinants of bird diversity and species composition of the bird communities. We recorded 6522 individual birds belonging to 146 species, 77 genera and 23 families. Resident birds made up 80% (117 species) of the total dataset. The study supported the original expectation that altitude is a major determinant of species richness and composition of bird communities in the area. More diverse bird communities were found also in areas with steeper slopes. This together with the positive effect of greater heterogeneity suggests that forests on steep slopes intermixed with patches of open habitats on shallow soil at large spatial scales are more important for diverse bird communities than more disturbed habitats on shallow slopes. In addition, we demonstrated that while different habitat characteristics such as presence of forests edges and shrubs play an important role in driving species composition, but they do not affect species richness. This indicates that while habitat conditions are important determinants of the distribution of specific species, the number of niches is determined by large scale characteristics, such as landscape level habitat heterogeneity and altitude. Thus, to protect bird diversity in the mid-hills of central Nepal, we should maintain diverse local habitats (viz. forest, shrubs, open land, etc.) but also make sure the natural habitats on steeper slopes with large scale heterogeneity are maintained.

## Introduction

Birds are an important part of ecosystems and a key part of food chains. For example, they eat insects, pollinate plants and disperse seeds [1]. Birds are also indicators of the quality of forest habitats [2] as they respond to habitat structure [3] and belong to several trophic guilds [4]. The distribution of many species of birds is affected by habitat fragmentation and reflects inter-specific dynamics and population trends associated with habitats [5]. Bird communities can be used to indicate the quality of habitats and thus can help guide management at regional and landscape levels [5,6].

Many recent studies focus on the distribution of bird species richness and diversity and their changes over time in various regions [7–10]. They show that bird diversity and richness are associated with presence of field margins [11–13], forest edges [14], habitat fragmentation [15], habitat quality [16], landscape changes [17], landscape structure and farming systems [13,18,19], type of vegetation [13,20] and climate [21]. A study of the global patterns in bird diversity [22] indicates that bird diversity on mountains in high rainfall regions decreases with altitude whereas on mountains in dry areas it is unimodal.

Although bird diversity and distribution are well studied in Europe [23–26] and America [27,28], there are very few similar studies for Asia in general [29] or the Himalayan region [30–34] and in particular Nepal [35–39]. Exploring the determinants of diversity in the Himalayas is important as in that region is the greatest variation in altitude anywhere in the world (i.e., 60 m to 8848 m) [9]. In a recent checklist for Nepal, a total of 871 species of birds are recorded including nine that are legally protected by the government of Nepal, 37 species that are globally threatened and 149 species that are nationally threatened [40]. It is thus considered as one of the most important places in the world for studying patterns in the distribution and diversity of species along altitudinal gradients [41]. The distribution of birds in the Himalayan region is associated with climatic factors (temperature, precipitation, seasons, area of landmasses, etc.) [42], and various kinds of anthropogenic activities, such as forest encroachment, livestock grazing, over extraction of forest products, forest fires, etc.

The most vulnerable areas in Nepal are low lying areas in the Terai, Siwalik Hills, Mahabharat and its valleys. These areas are densely populated [43] with high levels of anthropogenic activity, such as slash and burn cultivation, livestock grazing, habitat encroachment, etc. To properly protect the diversity of birds in these areas, it is important to understand the factors that determine the distribution of birds in the mid-hills of Nepal. There are very few protected areas in this region, even though it has a rich biota [9,44]. It is expected that such a study will help in determining the ecology of threatened species of birds in this region (e.g., [45,46]). Therefore, this study is designed to explore the factors affecting diversity and distribution of birds on different forested hills in Nepal. Specifically, we attempted to answer the following questions: (1) What is the pattern of distribution of birds in hilly regions in Nepal? and (2) What are the factors influencing the distribution of the birds? In order to answer the above questions, data on the composition of bird communities were collected along an altitudinal gradient at four places in two valleys in central Nepal. We also collected data on a range of environmental variables at all sites in order to determine their association with bird diversity and species composition of bird communities.

## Methods

### Ethics statement

This study was carried out in fully or partly managed community forests outside protected areas. We obtained permission for studying birds from different village development committees, the Dovan Khola Community Forest user groups (for the Kot-thumki forest) in Tistung, the Daman village development committee for Simbhanjyang, the Thankot village development committee for Chandragiri Sachet Mahila Community forest in Chandragiri and the Godawari village development committee for the Phulchowki community forest. This study did not involve the collection of any endangered or protected species. We recorded the species and counted the numbers of birds from a distance so specific permission was not required.

### Study area

**Fig 1:**
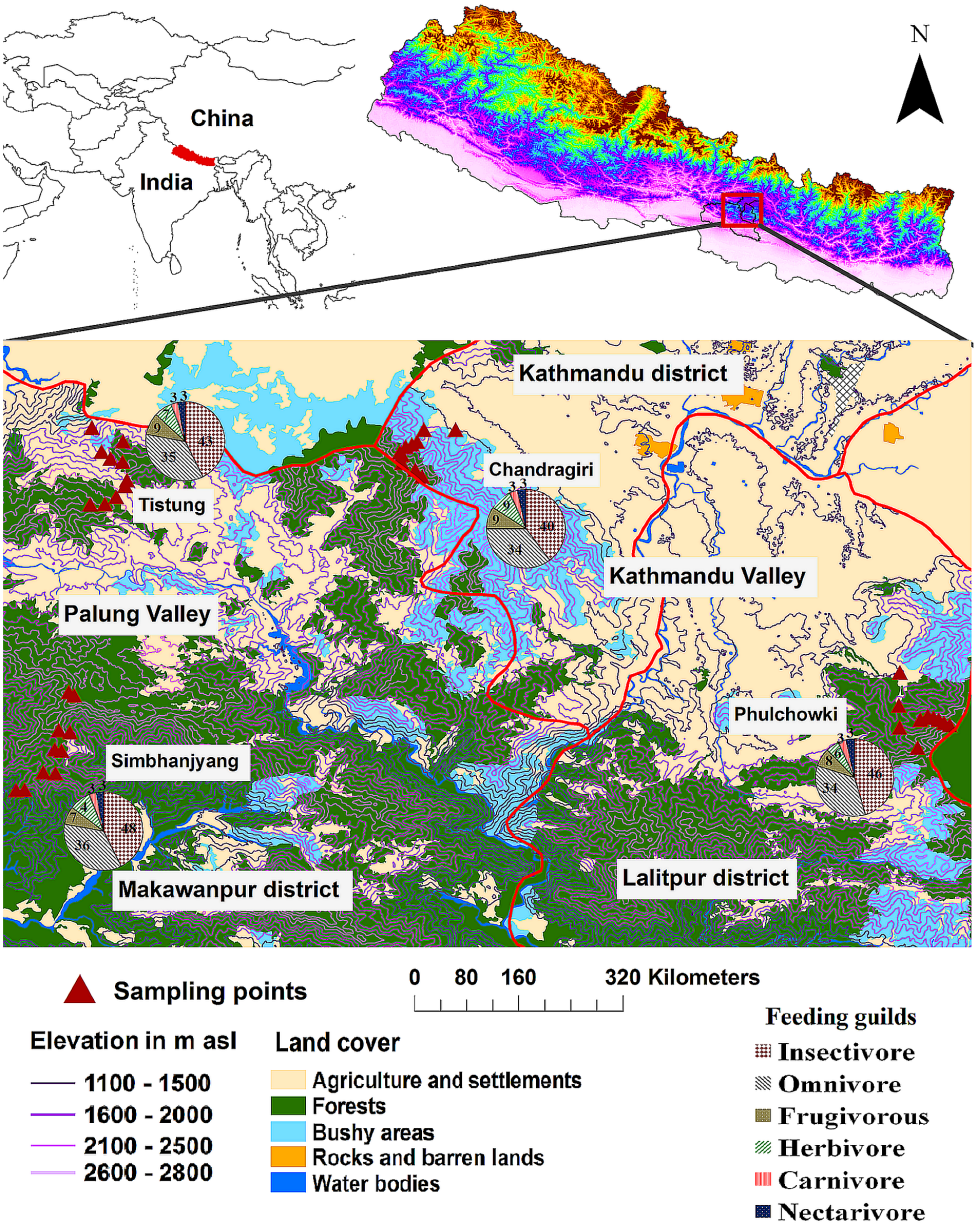
Maps showing the location of the study area and land use in the Palung and Kathmandu valleys, Nepal, along with pie-charts recording bird diversity in terms of feeding habits.

The study areas were located in the central mid-hills of the Himalayas in Nepal, which are located in two valleys, each with two localities. Two localities were in the Palung valley (Tistung, 27°39'N-85°05'E and Simbhanjyang, 27°33N-85°04'E) and two in the Kathmandu valley (Chandragiri, 27°40'N 85°12'E and Phulchowki, 27°34'N-85°23'E) in Nepal (Fig 1). Phulchowki hill is an Important Bird Area (IBA) and one of the 27 IBAs in Nepal (see [47]. An IBA is an area recognized as being highly important for the conservation of birds. Tistung and Simbhaynjyang are partially managed by a community forest and Chandragiri is fully managed by a community. Local people in community forest user groups are partially supervised by district forest offices and manage the natural resources of forested areas at particular locations. In both valleys 80% of the annual precipitation (about 1950 mm at Palung and 1639 mm at Kathmandu) falls during the monsoon season (June-September). However, short showers are common throughout the year. During January-February, snowfall is common 2300 meters above sea level (m a.s.l.) in both valleys. Kathmandu valley is between 1300 m and 2760 m a.s.l. and Makwanpur valley between 950 and 2582 m a.s.l. The average monthly maximum and minimum temperatures range between 14.8 and 2.4°C in winter and 22.3 and 14°C in summer at Palung, and 17.5 and 4.1°C in winter and 25.8 and 18.3°C in summer at Kathmandu, respectively [48]. Both these valleys are densely populated and subsistence agriculture is common at lower altitudes (between 1500 and 1700 m a.s.l. at Chandragiri and Tistung) and a major part of local livelihood. Forests are severely affected by human intervention in all the areas studied [49].

The vegetation in the area studied ranges from subtropical to temperate lower mixed broad-leaved forest. The subtropical vegetation is dominated by *Schima*-*Castanopsis*, Chir pine and alder forest. The lower temperate mixed broad-leaved forest is dominated by species of broad leaved trees of the genus *Quercus* mixed with abundant Laurels (*Lidera neesina* and *Litsea cubiba*). The dominant genera of shrubs are *Jasminum*, *Rubus*, *Viburnum*, *Eurya*, *Mahonia*, along with clumps of the bamboo, *Arundinaria falcata* [50–52].

### Data collection

Data were collected in two seasons in the same year (2009), which by including both pre breeding (April-early May) and post breeding (late May-June) seasons maximizes the chances of recording early breeding resident and late breeding migrant species. The birds present between 1500 m and 2400 m a.s.l. were recorded on the northern side of the mountains at four localities in both valleys because they are wetter than the south-facing slopes and have luxuriant vegetation suitable for birds [53]. The sampling was done every 100 m, with records collected at ten altitudes at each locality. There was one fixed circle at each altitude. Each circle had a 50 m radius. Thus, all together the birds observed in 10 circles each with a 50 m radius at each locality on each sampling occasion were recorded (i.e. 10 circles x 4 localities x 2 times = 80 circles in total).

The sampling was done in subtropical forest, shrubs, soil cliff and rocky area. As all sampling sites were chosen far from areas of human settlement, we completely excluded agricultural fields, orchards gardens and houses. Sampling at Chandragiri, Phulchowki and Tistung started at the edges of the forest and at Simbhanjyang in the forest in order to avoid settlements.

The locations of these circles were fixed with the help of an altimeter, Garmin GPS and a topographic map. Bird counting started normally early in the morning and continued to mid-morning each day. During data collection, three minutes were spent adjusting to the location and the next 20 minutes to counting birds in each circle at a particular altitude using the fixed point counting method [26,54–56]. We used a two man direct observation technique and vocal recording to identify and count the number of species of birds [57–59] using 10 x 42 mm binoculars plus recordings of their songs for verifying the canopy dwelling species [60]. In this study, we did not include high flying species like soaring raptors, swifts and swallows because of the difficulty of identifying and attributing an altitude.

Birds were identified in the field with the help of standard bird guide books [61,62]. Breeding birds were later confirmed with the help of additional literature [52,61–66]. Their conservation status was obtained from books [67] and online databases [68,69]. During the bird count, we also recorded the altitude (in meters above sea level), slope (angle between the horizontal line and inclination of the place in degrees), proportional canopy cover of forest, presence of litter or forest humus and forest and shrubby areas in each of the circles sampled (in proportions). The proportion of the area covered by forest canopy was visually estimated. Altitude was measured with an altimeter and slope with a compass clinometer. Presence or absence of forest, shrubby areas, litter and forest edge, in each circle sampled, were visually recorded. Presence was given a value of one and absence as zero. In our study, 'forest' was precisely classified as an area of land covered with trees or other woody vegetation and 'shrubs' was classified as a land covered with medium sized woody plants with multiple stems and usually under 6 meters tall [70]. The time of sampling was recorded as either time 1 (pre breeding, April-early May) or time 2 (post breeding season, late May-June).

We classified different bird guilds on the basis of their feeding habits [62]. They were insectivorous, omnivorous, frugivorous, herbivorous, carnivorous and nectivorous. We analyzed differences in the proportions of insectivorous, omnivorous and frugivorous birds as described below. The other guilds (herbivorous = 7 species, carnivorous = 6 species and nectivorous = 2 species) were not tested due to their low occurrence in the dataset.

### Data analysis

To assess the effect of landscape heterogeneity on bird distribution and different feeding guilds of birds, we scanned maps of the area (1:125000) and calculated length of all the edges between different habitats within a 100 m circle around each sampling point using NIS software. Habitat categories on the map were forested area, open places and water bodies. However, there were no agricultural fields, orchards gardens and houses as our sampling sites were far from settlements. We then used this data to calculate the weighted edge density according to Hargis et al. [71].

To assess the effect of spatial position of each sampling point on species richness and species composition and proportions of insectivorous, omnivorous and frugivorous birds, we calculated the Euclidean distance between all pairs of points using either the data on species composition or species richness. Species richness is defined as number of bird species present in a plot and species composition as the percentage (%) of various bird species in relation to the total in a given area. We also calculated geographic distances between all pairs of plots. We used the Mantel test as implemented in the package Vegan in R with 1000 permutations to calculate the correlation between geographic distance and distance in species richness and composition. In the case of a significant effect of geographic distance, geographic position of the sampling points was used as a covariate in the subsequent analyses.

To identify the determinants of bird species richness, we used a generalized linear mixed effect model (GLMER) with Poisson distribution and log link function. The analyses were carried out using LME4 package in R [72]. The figures were drawn using STATISTICA [73]. Specifically, we tested the effect of altitude and the following local habitat characteristics: slope, forest canopy, shrub canopy, time of sampling, presence/absence of forest edge, presence/absence of litter and landscape heterogeneity. Locality and when sampled were used as random factors in the models. Significant habitat characteristics were selected using a forward step-wise selection procedure. Inclusion of a term into the model was assessed using the Akaike information criterion (AIC) as implemented in R [74].

We used a similar GLMER model to study the effect of habitat characteristics on occurrence of different feeding guilds. We linked the number of insectivorous and non-insectivorous birds at each location using a c-bind function and tested the effect of the predictors assuming binomial distribution of the dependent variable. Similarly, we tested the effects of predictors on the proportions of omnivorous and frugivorous birds. The results thus indicate the effects of different habitat characteristics on proportion of these guilds in the bird community.

Multivariate tests of species composition were carried out using a unimodal technique because we have only presence/absence data [75] and the gradient length was quite long (3.35). Therefore, we used Canonical Correspondence Analysis (CCA) to show the relationship between bird species and environmental variables. The significance of the predictors was tested using a Monte Carlo permutation test. All tests were carried out using Canoco 5.01 [76]. For these data, we used locality and recording time as covariates and tested the effect of altitude, and the local habitat characteristics: slope, forest canopy, shrub canopy, time of sampling, presence/absence of forest edge, presence/absence of litter and landscape heterogeneity. Significant habitat characteristics were selected using a forward step-wise selection procedure [75].

## Results

### Diversity and conservation status

Altogether 6522 birds (Chandragiri-1822, Phulchowki-1816, Shimbhanjyang-1556 and Tistung-1328) were recorded at all the sites. This included 146 species of birds belonging to 77 genera, 23 families and eight orders (S1 Appendix). The highest numbers recorded at all sites were of insectivorous followed by omnivorous and frugivorous birds. The rarest birds were nectarivorous (Fig 1). Eighty percent of the birds (117 species) recorded were residents and the remaining 20% (29 species) were migratory species. The largest family in terms of the number of species recorded was the Sylviidae with 37 species, followed by Muscicapidae with 35 species and Corvidae with 18 species.

*Turdoides nipalensis*, a species endemic to Nepal, was recorded at Chandragiri. Three species of birds in the national threatened category were recorded in the study areas. Among them, one endangered species *Brachypteryx leucophrys* was recorded at Phulchowki. Likewise, two vulnerable species were recorded, *Cutia nipalensis* at Simbhanjyang and *Garrulax caerulatus* at Phulchowki. In addition, we recorded five species of birds protected under CITES Appendix II. There were three of these species at Phulchowki, two at Chandragiri and Simbhanjyang and one at Tistung (S1 Appendix).

We recorded 119 species in the Kathmandu valley (Phulchowki—94 species and Chandragiri—88) and 99 species in the Palung valley (Shimbhanjyang—75 species and Tistung—68). In total, 47 species were only recorded in the Kathmandu valley (22 at Phulchowki and 15 at Chandragiri) and 27 species only in the Palung valley (13 at Shimbhanjyang and eight at Tistung).

### Effect of space

The Mantel test revealed a significant correlation between geographic distance and species richness recorded at the sampling points (r = 0.123, p = 0.003). In contrast, species composition of the birds recorded at the sampling points is independent of geographic position (r = 0.004, p = 0.439). Proportion of insectivorous (r = 0.19, p < 0.001) and frugivorous (r = 0.21, p < 0.001), but not omnivorous (r = 0.03, p = 0.19) was affected by spatial position of the sites. We thus included longitude, latitude and their interaction into the tests predicting species richness and proportion of insectivorous and omnivorous birds (Table 1).

**Table 1:** The association between species richness, proportion of omnivorous, frugivorous and insectivorous species and species composition, altitude and habitat characteristics. For species richness and proportions of the different feeding guilds, locality and time of sampling (pre- or post-breeding) were used as random effects in the models and tested using GLMER. For species composition, locality and time of sampling were used as covariates and the associations determined using CCA. Longitude, latitude and their interaction were used a covariates in cases of a significant association with space on the given dependent variables as identified by a Mantel test. Only variables selected as significant by a step wise selection are presented.

	Species richness		Proportion of omnivorous species		Proportion of frugivorous species		Proportion of insectivorous species		Species composition	
	Dev.	*P*-value	Dev.	*P*-value	Dev.	*P*-value	Dev.	*P*-value	*p-value*	% explained
Longitude	9.493	0.002	-	-	12.326	<0.001	19.059	<0.001	-	-
Latitude	1.133	0.287	-	-	3.815	0.051	7.151	0.007	-	-
Longitude x Latitude	7.53	0.006	-	-	-	-	-	-	-	-
Altitude	45.22	<0.001	-	-	17.708	<0.001	5.268	0.022	0.002	2.1
Heterogeneity	4.068	0.047	5.935	0.015	-	-	-	-	-	-
Canopy	-	-	-	-	-	-	-	-	0.024	1.6
Forest edge	-	-	-	-	-	-	-	-	0.002	3.1
Slope	4.799	0.036	-	-	-	-	-	-	0.028	1.63
Litter content	-	-	-	-	-	-	-	-	-	-
Shrubs	-	-	-	-	-	-	-	-	0.022	5.2
Forest	-	-	-	-	-	-	-	-	-	-

### Species richness

**Fig 2:**
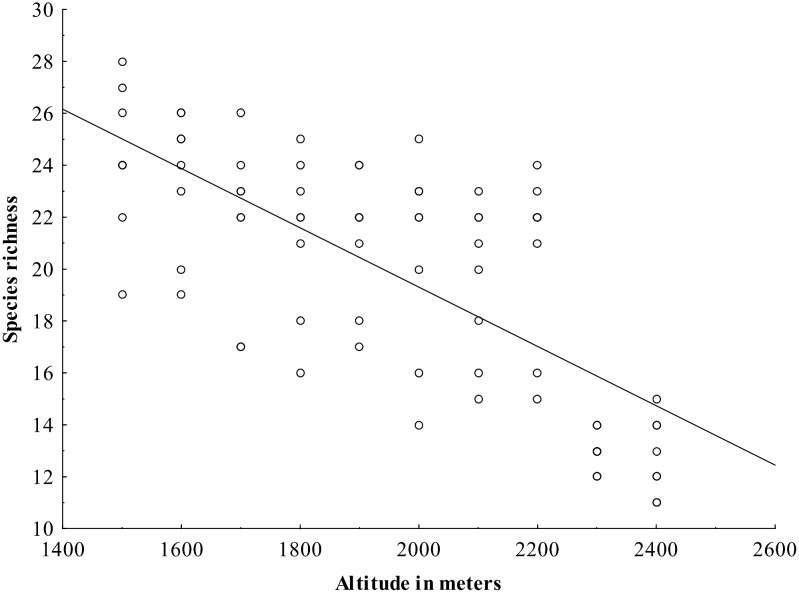
Relationship between bird species richness and altitude in central Nepal.

**Fig 3:**
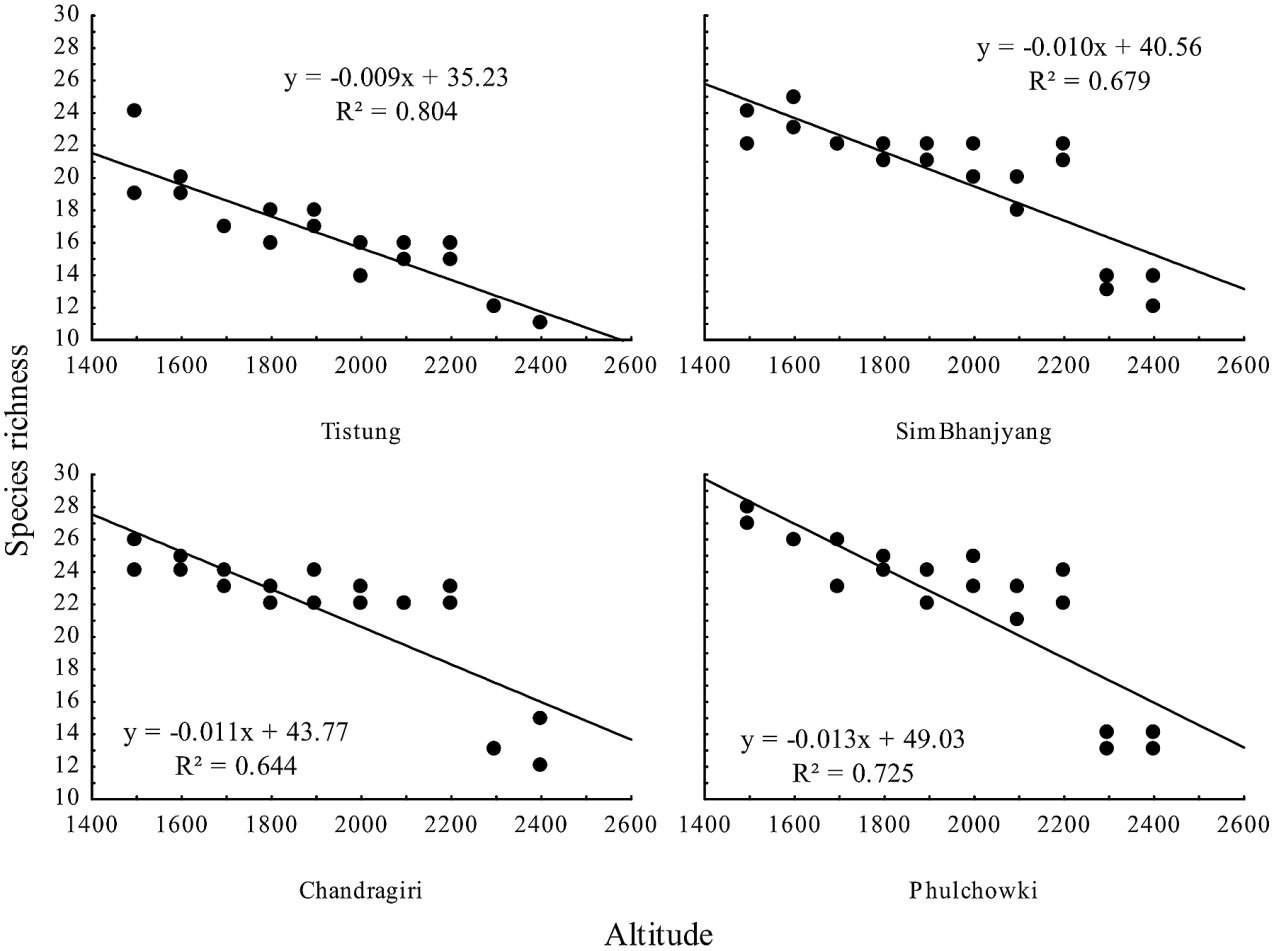
Relationship between species richness and altitude at different localities in central Nepal.

**Fig 4:**
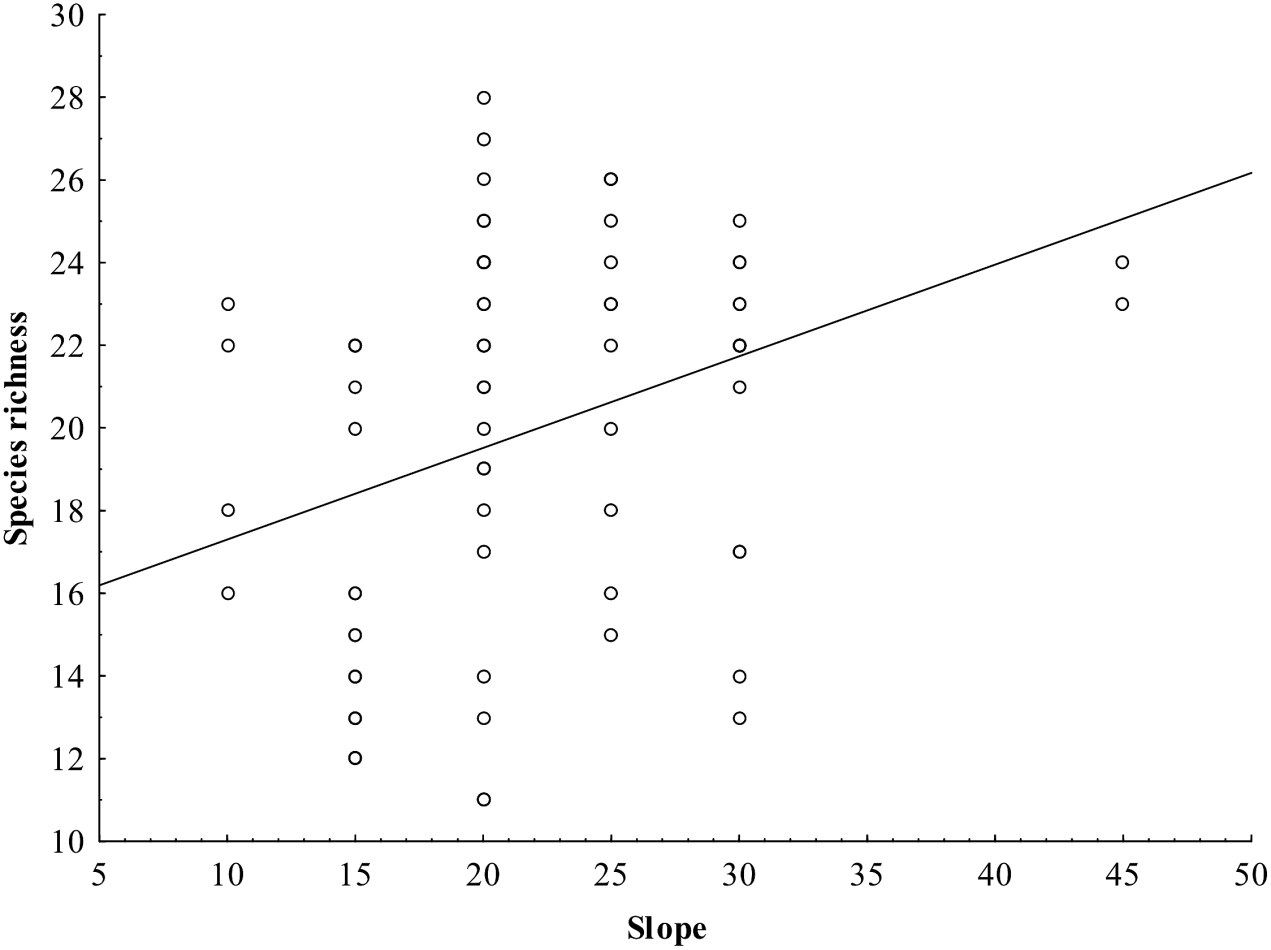
Relationship between species richness and slope at different localities in central Nepal. The relationship was significant even after removing outlying points with slopes of over 30 degrees (30 and 45).

Bird species richness significantly decreased with increasing altitude in the overall dataset (p <0.001, R^2^ = 0.5193) (Fig 2, S2 Appendix) as well as at particular localities (Table 1, Fig 3, S3 Appendix). Species richness also increased with slope (Fig 4), even when the two outlying points with slopes of over 30 degrees (30 and 45) were removed (p = 0.001, R^2^ = 0.150), and with increasing heterogeneity of the habitat (Table 1). No other habitat characteristic was significant (Table 1).

### Proportions of different guilds

**Fig 5:**
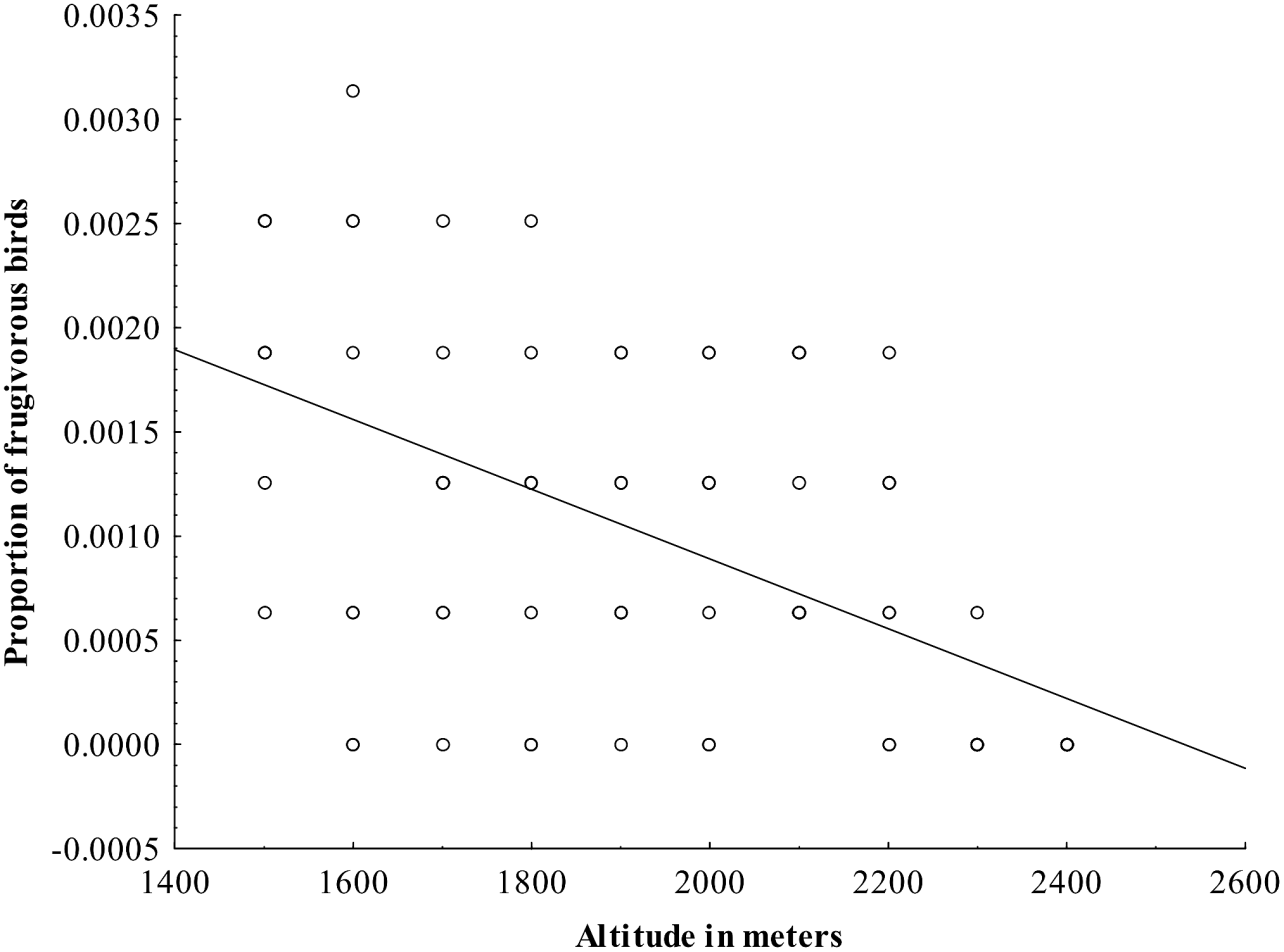
The relationship between the proportion of frugivorous bird species and altitude.

**Fig 6:**
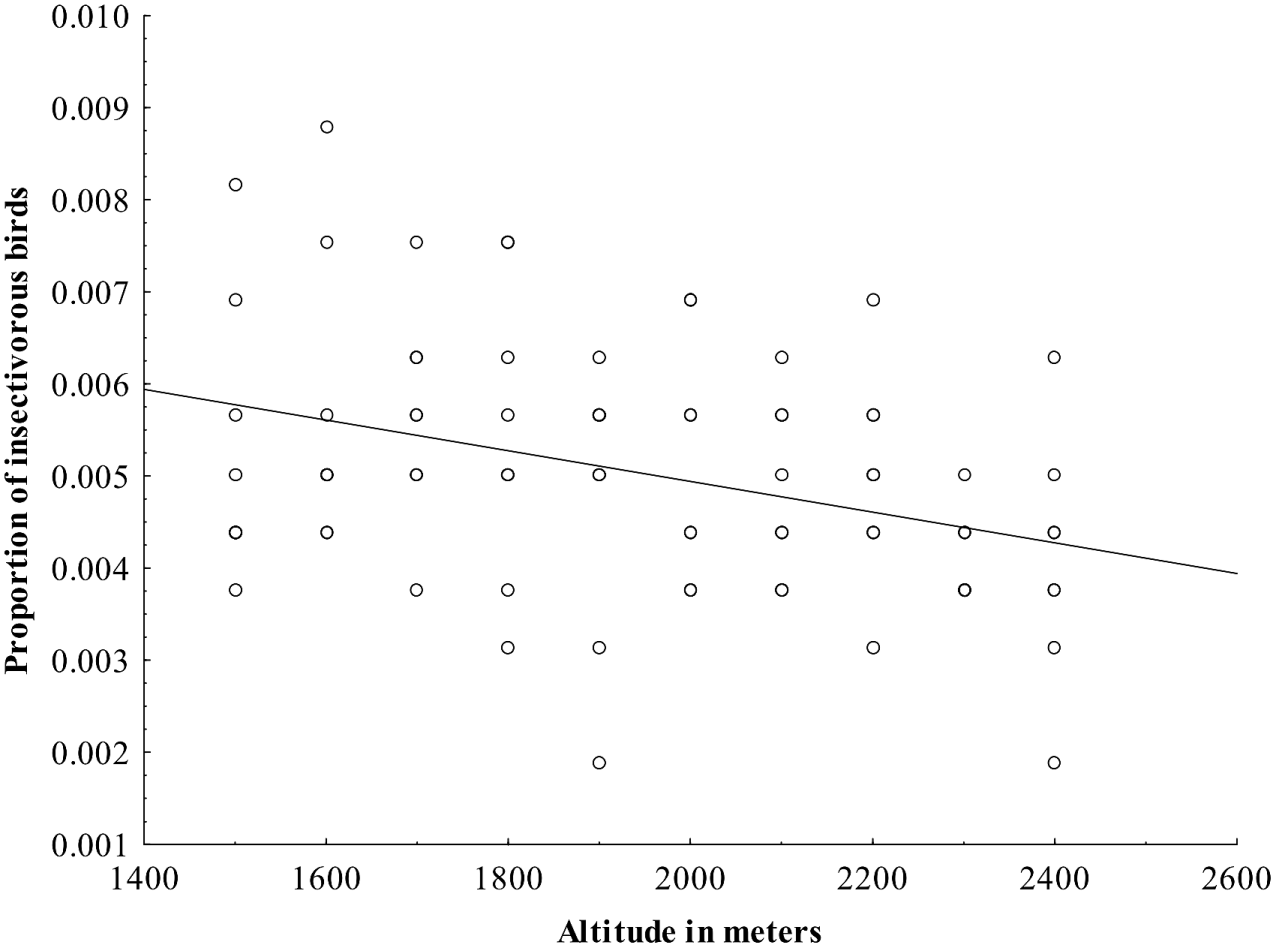
The relationship between the proportion of insectivorous bird species and altitude.

From the analysis on the effect of habitat characteristics on occurrence of different feeding guilds, it was found that the proportion of omnivorous species increased with increasing heterogeneity and was independent of any other habitat characteristics. In contrast, the proportions of frugivorous (Fig 5) and insectivorous birds (Fig 6) decreased with increasing altitude and was independent of any other habitat characteristics (Table 1).

### Species composition

**Fig 7:**
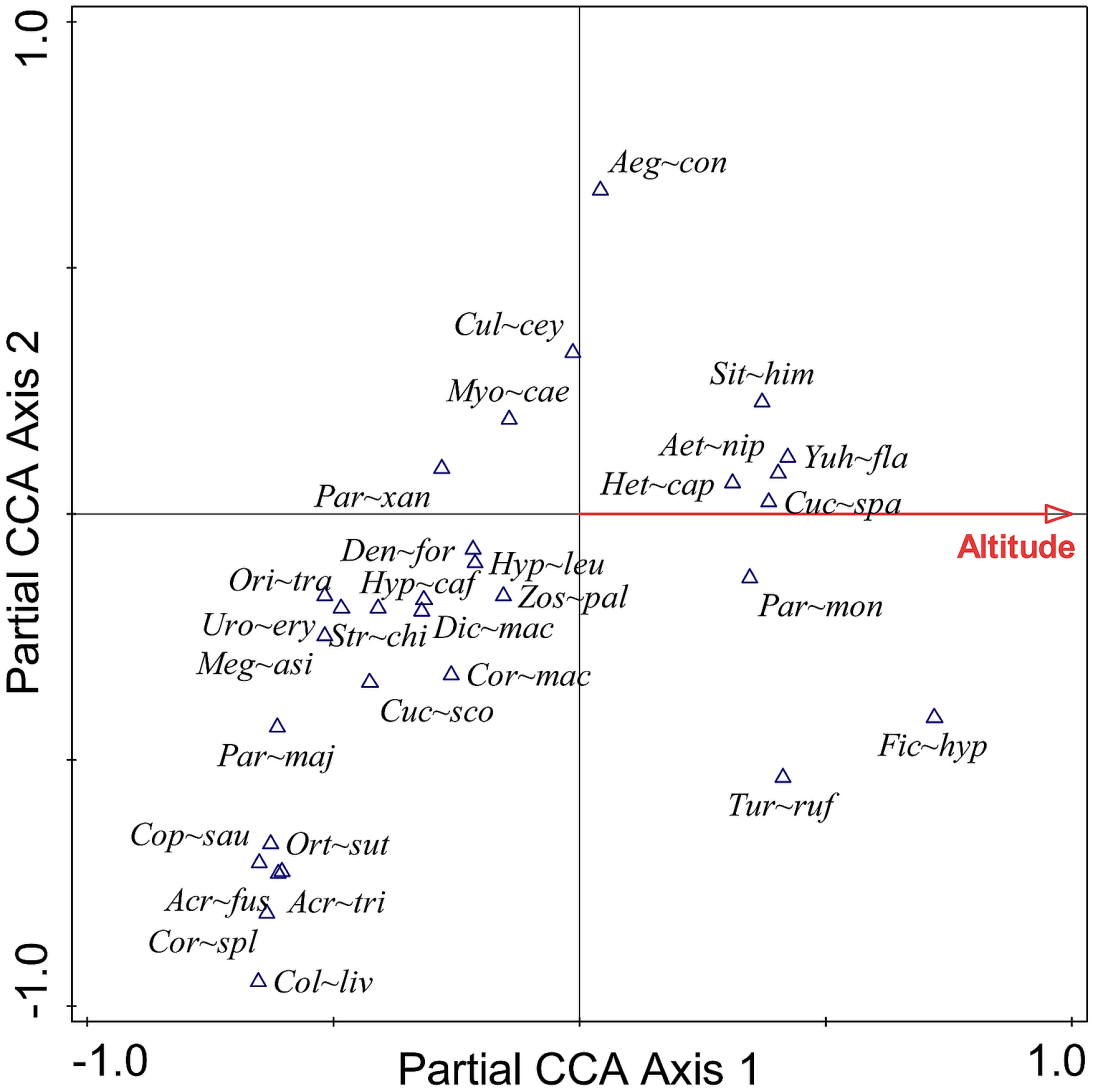
Relationship between the distribution of birds and altitude. The first axis explains 10.52% and the second axis explains 8.22% of the total variation in the dataset. For details of species name see S1 Appendix.

Species composition was significantly associated with altitude. The figure for those species of birds that occurred frequently indicates that the species recorded at the highest altitudes were *Hierococcyx sparverioides*, *Yuhina flavicollis*, *Aethopyga nipalensis*, *Heterophasia capistrata*, *Parus monticolus* and *Sitta himalayensis*. Species such as *Oriolus traillii*, *Urocissa erythrorhyncha* and *Megalaima asiatica* prevailed at the lowest altitude (Fig 7).

**Fig 8:**
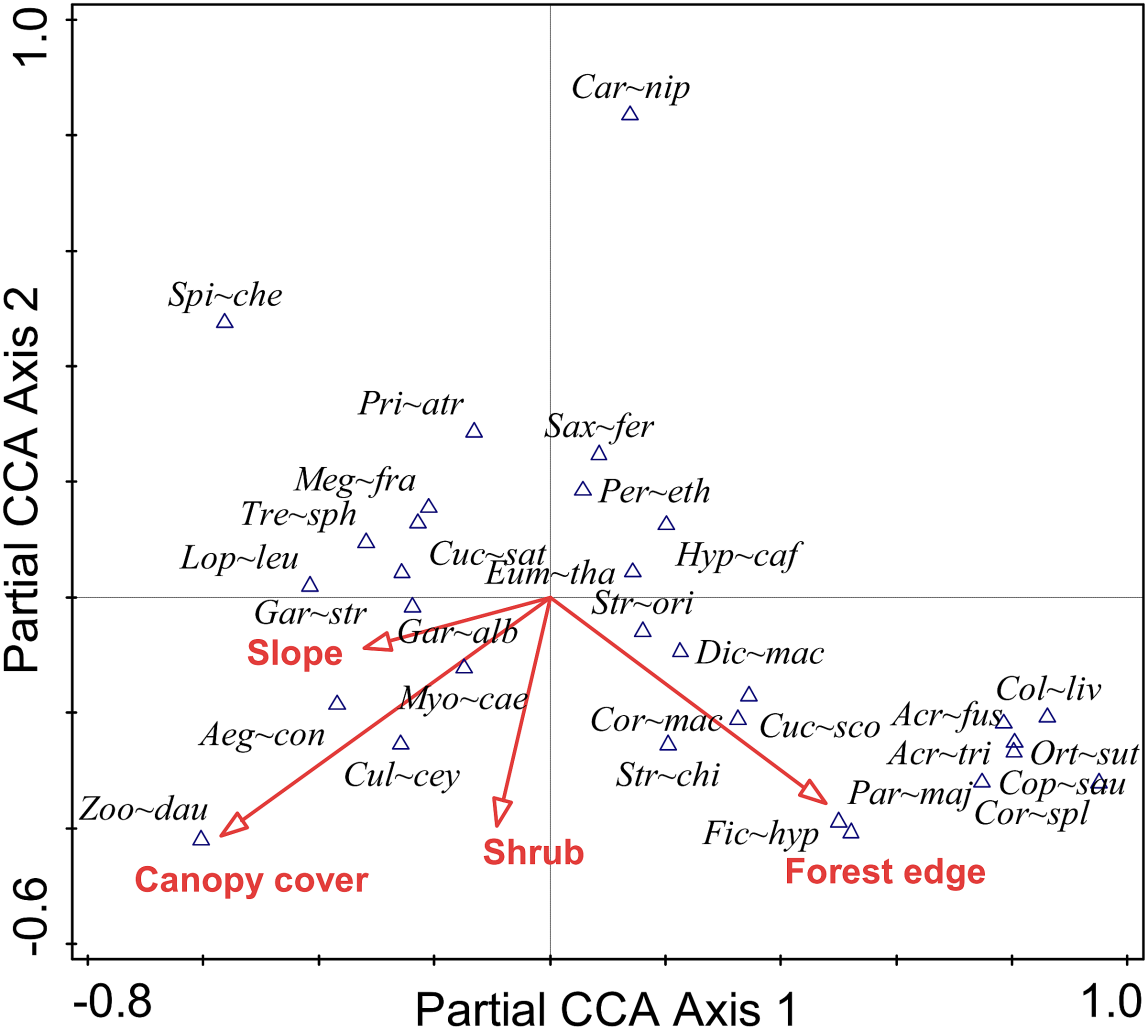
Relationship between the distribution of birds and different environmental factors. The first axis explains 4.98% and the second axis explains 2.91% of the total variation in the dataset. For details of species name see S1 Appendix.

Apart from altitude, four different environmental variables (canopy, forest edge, slope and shrubby area) were significantly associated with the species composition of bird communities in central Nepal. Together, they accounted for 11.1% of the total variation in the dataset (Table 1). *Parus major*, *Ficedula hyperythra* and *Copsychus saularis* preferred forest edges whereas *Spilornis cheela*, *Prinia atrogularis*, *Megalaima franklinii* and *Treron sphenura* preferred the forested areas. *Garrulax albogularis* and *Garrulax striatus* were recorded on steep slopes and *Heterophasia capistrata*, *Pericrocotus ethologus* in flat places. *Zoothera daum*, *Aegithalos concinnus* and *Culicicapa ceylonensis* preferred forest with a dense canopy. *Myophonus caeruleus and Culicicapa ceylonensis* were recorded in places where shrubs were abundant whereas *Eumyias thalassina*, *Pericrocotus ethologus*, *Heterophasia capistrata*, *Saxicola ferrea* and *Carpodacus nipalensis* were recorded in open areas (Fig 8).

## Discussion

### Diversity and conservation status

This study aimed at finding the factors affecting the distribution of birds in different places in central Nepal and provides crucial background data for identifying important bird rich areas. In turn, it will indirectly help in formulating management plans and also in defining priority areas for bird conservation. Our study indicates that the Kathmandu valley is richer in birds than the Palung valley. At Phulchowki, however, we recorded one globally near-threatened and nationally critically endangered species, the Laggar Falcon (*Falco jugger*). There are also nationally endangered species at this site such as the Red-headed Trogon (*Harpactes erythrocephalus*), Blue-naped Pitta (*Pitta nipalensis*), White-throated Bulbul (*Alophoixus flaveolus*) and Purple Cochoa (*Cochoa purpurea*), as reported by Baral and Inskipp [47]. The number of important species of birds recorded at Phulchowki well justifies its important bird area (IBA) status [47].

The Nepalese endemic species, Spiny babbler (*Turdoides nipalensis*), was recorded at Chandragiri, as reported in a previous study [52]. Although a road was constructed in this area, which resulted in a recent increase in number of visitors, Chandragiri still remains a very important habitat for birds due to presence of thick forest on its north facing slopes.

Compared to the number of species of birds recorded for the whole of Nepal (877 bird species) [33], the number of endangered species reported in this study is quite low because most of the endangered species of birds in Nepal occur at either low altitudes or in high altitudinal zones and this study focused on intermediate altitudinal zones [47,65,77]. The low number of endangered birds could also partly be a result of our sampling technique, which was not intensive enough to record species that are very rare. In addition to this, as rare species are very elusive in nature, more effort is needed to spot them the field. Thus, the point count sampling technique used in this study is not appropriate for recording rare species [78].

Insectivore birds were the dominant and carnivores the rarest guild recorded in this study, which is similar to figures for the total bird species recorded in Nepal [40,77] and other areas [79,80]. Another reason for recording a very high proportion of insectivorous birds may be the exclusion of different soaring raptors and other high flying birds such as swallows (*Hirundo* spp.), martins (*Delichon* sp.) and crows (*Corvus* spp.) due to the difficulty of identifying the exact species. The family patterns of the birds recorded in this study is comparable to that for total bird species of Nepal [40,77].

### Effects of space

The correlations of species richness and proportion of frugivorous and insectivorous species with geographic distances indicate that the distribution of suitable habitats is largely spatially autocorrelated and that neighbouring localities are more likely to have a similar species richness and feeding guilds. The fact that spatial effects for different feeding guilds were detected but not for species composition, may indicate that different species from the same guilds may replace each other in neighbouring localities and the exact species composition is determined more by local habitat conditions. We should, however, be cautious about accepting this conclusion as the number of birds recorded at each sampling point was relatively low and the high turnover in species composition may be also at least partly caused by low sampling effort. Significant effect of longitude and latitude, which were included as covariates in the subsequent tests supported the importance of space for these variables. These variables can in fact act as surrogates of climatic variables at larger spatial scales [81]. In contrast to our results, the geographic variables (longitude and latitude) are regarded as important determinants not only of species richness [82] but also of the occurrence of specific birds and thus of species composition in several previous studies (e.g. [83,84]), which indicates that spatially autocorrelated edaphic and floristic differences are the main factors driving this pattern. These studies were, however, done in areas with a less variable topography and where large scale differences are likely to dominate over local habitat differentiation.

### Species richness and composition

It is previously reported that distributions of birds are determined by different environmental factors such as floristic composition, habitat structure, food availability, temperature and climate [85–88]. The inverse relationship of species richness and altitude in two valleys (the Palung and Kathmandu valleys) in central Nepal is similar to the patterns recorded in previous studies on birds in the Himalayan region [9,32,34] and the world [22]. The sparseness of the vegetation at high altitudes due to the stressful climate and poor food resources are likely to be the main reason for low bird diversity [9,89,90]. As indicated by the species-area relationship, the available area in hilly or mountain areas at high altitudes decreases compared to low altitudes because of mountain structures. So, when considering species-area relationships, more species are expected at low altitudes due to the presence there of large areas suitable for species [9,91]. An alternative explanation for the decrease in species richness with increase in altitude could also be due to the distributional non-overlap [92] associated with the radical change in habitats with altitude in different places.

Altitude is strongly associated with not only species richness but also the distribution of the different feeding guilds and composition of bird communities, both in our study and also a range of previous studies [8,22,93,94]. Specifically, the proportion of frugivorous and insectivorous birds decreased with increasing altitude in our study while proportion of omnivorous birds was not associated with altitude. Thus, insectivorous birds are more associated with places at low altitudes where the vegetation is dense. The number of nectarivorous and herbivorous species recorded in this study, however, was low and not tested statistically. These patterns are comparable to those recorded in other areas, such as in [93], and on Mount Kilimanjaro, Tanzania [8] and [95]. The association of frugivorous birds in lower altitudes compared to higher is also due to the presence of more trees with fleshy fruits which provide them abundant food resources [96].

The present study also revealed that bird richness was higher on steeper slopes and in more heterogeneous habitats. The high species richness recorded on steep slopes could be linked to the fact that communities on steep slopes are better conserved compared to those in flat areas as people cannot easily access them for agriculture or settlement or fetching forest resources such as firewood, fruits, etc. The positive effect of heterogeneity on species richness of birds is in line with that reported in several previous studies [97–100]. It can be explained in terms of the 'habitat heterogeneity hypothesis', which suggests that heterogeneous habitats provide more niches and diverse ways of exploiting the environmental resources (see Tews et al. for review) [101] and thus increasing species diversity [85,102,103]. This is also in accordance with predictions of the species richness-energy hypothesis [104]. A similar effect was recorded for omnivorous birds but not for any other feeding guild. This is probably because omnivorous birds are able to utilize a wide range of resources and are efficient at utilizing environments with diverse food sources. The absence of any effect on the other feeding guilds can be explained by the fact that specific feeding guilds are confined to their own habitat and habitat heterogeneity may in fact lower the availability of each specific resource. While previous studies demonstrate that other habitat characteristics, such as forest fragmentation and agricultural activity, affect the richness of different feeding guilds [105,106] no such effects were recorded in this study. This is likely to be due to the fact that a major portion of the variation in the different feeding guilds is associated with altitude and space, which prevents other effects being significant.

The species richness, feeding guilds and species composition of bird communities differed in the two valleys. In contrast to feeding guilds and partly also to species richness, species composition was strongly associated with environmental variables such as percentage canopy cover, presence of forest edge, slope and shrubs. The major reason for this is the association of specific types of birds with particular types of habitats due to the availability of shelter and food. Of these variables, forest edges seem to be the most important as they provide specific habitats for some species of birds while other species are associated with resources found within highly productive woodlands and tend to avoid forest edges [107]. Species such as Golden-throated Barbet (*Megalaima franklinii*), Oriental Cuckoo (*Cuculus saturatus*), Wedge-tailed Green Pigeon (*Treron sphenura*), Crested Serpent Eagle (*Spilornis cheela*), etc. are restricted to the forests in these mountains. Thus, the habitat specificity and feeding habits of some birds are the major reasons for the association of bird communities in central Nepal with different environmental variables. Interestingly, factors strongly associated with species composition are not associated with species richness, indicating that habitat characteristics associated with occurrence of specific species are not associated with the number of niches available.

## Conclusions

The results of this study support the expectation that altitude is a major determinant of species richness and composition of bird communities in areas that vary greatly in altitude and is strongly associated with the distribution of different feeding guilds within the area. The greatest diversities of birds were recorded on steep slopes with heterogeneous habitats, which provide many habitats for birds. In addition, we demonstrate that different habitat characteristics, such as forest edges and shrubs, are strongly associated at local spatial scales with the distributions of species, but not species richness. This indicates that while habitat conditions are important determinants of the distributions of specific species, the number of niches are more likely to be determined by larger scale characteristics such as landscape level habitat heterogeneity and altitude.

Thus, to protect bird diversity in the mid-hills of central Nepal, we should maintain there a diversity of habitats (viz. forest, shrubs, open land, etc.) and especially protect the heterogeneous habitats on steep slopes, as these habitats are the most species rich.

## Supporting Information

S1 Appendix List of all the species of birds recorded in this study with common names, feeding habit, seasonal status and threatened status. Presence of the species at only one of the localities studied is shown by + in the respective column.
(DOCX)

S2 Appendix Relationship between bird species richness and altitude in central Nepal determined using exponential line fitting. (TIF)

S3 Appendix Relationship between species richness and attitude at different localities in central Nepal determined using exponential line fitting. (TIF)

S1 Supporting Information Details of Accession Numbers. (DOCX)
