# Supplementary material for: Heterogeneous Landscapes on Steep Slopes at Low Altitudes as Hotspots of Bird Diversity in a Hilly Region of Nepal in the Central Himalayas
Source: PLoS One. 2016 Mar 3;11(3):e0150498. doi: 10.1371/journal.pone.0150498 (PMC4777546; doi:10.1371/journal.pone.0150498)
Supplement: S1 Appendix — Presence of the species at only one of the localities studied is shown by + in the respective column. (DOCX) [file pone.0150498.s001.docx]

**S1 Appendix** List of all the species of birds recorded in this study with common names, feeding habit, seasonal status and threatened status. Presence of the species at only one of the localities studied is shown by + in the respective column.

| **S.N.** | **Scientific name** | **Abbreviations** | **Common Name** | **Feeding habit** | **Seasonal status*** | **IUCN, NT & CITES**** | **Phulchowki** | **Chandragiri** | **Simbhanjyang** | **Tistung** |
| --- | --- | --- | --- | --- | --- | --- | --- | --- | --- | --- |
| 1 | *Abroscopus schisticeps* | Abr~sch | *Black-faced Warbler* | Omnivore | r | LC | + | + | + |  |
| 2 | *Acridotheres fuscus* | Acr~fus | *Jungle Myna* | Omnivore | r | LC | + | + |  |  |
| 3 | *Acridotheres tristis* | Acr~tri | *Common Myna* | Omnivore | r | LC | + | + |  |  |
| 4 | *Aegithalos concinnus* | Aeg~con | *Black-throated Tit* | Insectivore | r | LC | + | + | + | + |
| 5 | *Aegithalos niveogularis* | Aeg~niv | *White-throated Tit* | Insectivore | r | LC |  | + |  |  |
| 6 | *Aethopyga gouldiae* | Aet~gou | *Mrs Gould’s Sunbird* | Nectarivore | r | LC | + |  |  |  |
| 7 | *Aethopyga nipalensis* | Aet~nip | *Green-tailed Sunbird* | Nectarivore | r | LC | + | + | + |  |
| 8 | *Aethopyga siparaja* | Aet~sip | *Crimson Sunbird* | Nectarivore | r | LC |  | + |  | + |
| 9 | *Alcippe castaneceps* | Alc~cas | *Rufous-winged Fulvetta* | Insectivore | r | LC |  | + |  |  |
| 10 | *Alcippe nipalensis* | Alc~nip | *Nepal Fulvetta* | Insectivore | r | LC | + |  | + | + |
| 11 | *Alcippe vinipectus* | Alc~vin | *White-browed Fulvetta* | Insectivore | r | LC | + | + | + |  |
| 12 | *Anthus hodgsoni* | Ant~hod | *Olive-backed Pipit* | Insectivore | r | LC |  | + | + | + |
| 13 | *Anthus sylvanus* | Ant~syl | *Upland Pipit* | Insectivore | r | LC |  |  |  | + |
| 14 | *Ardeola grayii* | Ard~gra | *Indian Pond Heron* | Carnivore | r | LC | + |  |  |  |
| 15 | *Brachypteryx leucophrys* | Bra~leu | *Lesser Shortwing* | Insectivore | r | LC, En | + |  |  |  |
| 16 | *Brachypteryx montana* | Bra~mon | *White-browed Shortwing* | Insectivore | r | LC | + |  |  |  |
| 17 | *Carpodacus erythrinus* | Car~ery | *Common Rosefinch* | Herbivore | r | LC |  | + |  | + |
| 18 | *Carpodacus nipalensis* | Car~nip | *Dark-breasted Rosefinch* | Herbivore | r | LC |  | + |  |  |
| 19 | *Chaimarrornis leucocephalus* | Cha~leu | *White-capped Water Redstart* | Omnivore | r | LC |  |  | + | + |
| 20 | *Cissa chinensis* | Cis~chi | *Common Green Magpie* | Carnivore | r | LC | + |  |  |  |
| 21 | *Columba livia* | Col~liv | *Blue Rock Pigeon* | Herbivore | r | LC | + | + |  |  |
| 22 | *Copsychus saularis* | Cop~sau | *Oriental Magpie Robin* | Insectivore | r | LC | + | + |  | + |
| 23 | *Coracina macei* | Cor~mac | *Large Cuckooshrike* | Insectivore | r | LC |  | + | + |  |
| 24 | *Corvus macrorhynchos* | Cor~mac | *Large-billed Crow* | Omnivore | r | LC | + | + | + | + |
| 25 | *Corvus splendens* | Cor~spl | *House Crow* | Omnivore | r | LC | + | + |  |  |
| 26 | *Cuculus canorus* | Cuc~can | *Eurasian Cuckoo* | Insectivore | s | LC | + | + | + | + |
| 27 | *Cuculus micropterus* | Cuc~mic | *Indian Cuckoo* | Omnivore | s | LC |  | + |  |  |
| 28 | *Cuculus saturatus* | Cuc~sat | *Oriental Cuckoo* | Omnivore | s | LC | + | + | + | + |
| 29 | *Culicicapa ceylonensis* | Cul~cey | *Grey-headed Canary Flycatcher* | Insectivore | r | LC | + | + | + | + |
| 30 | *Cutia nipalensis* | Cut~nip | *Nepal Cutia* | Insectivore | r | LC, V |  |  | + |  |
| 31 | *Dendrocitta formosae* | Den~for | *Grey Treepie* | Omnivore | r | LC | + | + | + | + |
| 32 | *Dendrocitta vagabunda* | Den~vag | *Rufous Treepie* | frugivorous | r | LC |  | + |  | + |
| 33 | *Dendrocopos auriceps* | Den~aur | *Brown-fronted Woodpecker* | Omnivore | r | LC |  |  | + |  |
| 34 | *Dicaeum ignipectus* | Dic~ign | *Fire-breasted Flowerpecker* | Nectarivore | r | LC | + | + | + | + |
| 35 | *Dicrurus leucophaeus* | Dic~leu | *Ashy Drongo* | Insectivore | s | LC |  | + | + |  |
| 36 | *Dicrurus macrocercus* | Dic~mac | *Black Drongo* | Insectivore | r | LC | + | + | + | + |
| 37 | *Dicrurus remifer* | Dic~rem | *Lesser Racket-tailed Drongo* | Insectivore | s | LC | + |  | + |  |
| 38 | *Enicurus schistaceus* | Eni~sch | *Slaty-backed Forktail* | Insectivore | r | LC | + |  | + |  |
| 39 | *Eudynamys scolopacea* | Cuc~sco | *Asian Koel* | Omnivore | r | LC | + | + |  | + |
| 40 | *Eumyias thalassina* | Eum~tha | *Verditer Flycatcher* | Insectivore | s | LC | + | + | + | + |
| 41 | *Ficedula hyperythra* | Fic~hyp | *Snowy-browed Flycatcher* | Insectivore | r | LC | + |  |  | + |
| 42 | *Ficedula strophiata* | Fic~str | *Rufous-gorgeted Flycatcher* | Insectivore | r | LC |  |  |  | + |
| 43 | *Ficedula superciliaris* | Fic~sup | *Ultramarine Flycathcher* | Insectivore | s | LC | + |  |  |  |
| 44 | *Ficedula tricolor* | Fic~tri | *Slaty-blue Flycatcher* | Insectivore | r | LC |  | + |  |  |
| 45 | *Ficedula westermanni* | Fic~wes | *Little Pied Flycatcher* | Insectivore | r | LC |  |  | + |  |
| 46 | *Francolinus francolinus* | Fra~fra | *Black Francolin* | Herbivore | r | LC |  |  |  | + |
| 47 | *Garrulax albogularis* | Gar~alb | *White-throated Laughingthrush* | Omnivore | r | LC | + | + | + |  |
| 48 | *Garrulax caerulatus* | Gar~cae | *Grey-sided Laughingthrush* | Omnivore | r | LC, V | + |  |  |  |
| 49 | *Garrulax erythrocephalus* | Gar~ery | *Chestnut-crowned Laughingthrush* | Omnivore | r | LC |  | + |  |  |
| 50 | *Garrulax leucolophus* | Gar~leu | *White-crested Laughingthrush* | Omnivore | r | LC | + |  |  |  |
| 51 | *Garrulax lineatus* | Gar~lin | *Streak Laughigthrush* | Omnivore | r | LC | + | + | + | + |
| 52 | *Garrulax striatus* | Gar~str | *Striated Laughingthrush* | Omnivore | r | LC | + | + | + | + |
| 53 | *Garrulax variegatus* | Gar~var | *Variegated Laughingthrush* | Omnivore | r | LC | + | + | + |  |
| 54 | *Garrulus glandarius* | Gar~gla | *Eurasian Jay* | Herbivore | r | LC | + | + | + | + |
| 55 | *Garrulus lanceolatus* | Gar~lan | *Black-headed Jay* | Herbivore | r | LC | + |  |  |  |
| 56 | *Glaucidium cuculoides* | Gla~cuc | *Asian Barred Owlet* | Carnivore | r | LC, II | + |  | + |  |
| 57 | *Glaucidium radiatum* | Gla~rad | *Jungle Owlet* | Carnivore | r | LC, II |  |  | + |  |
| 58 | *Heterophasia capistrata* | Het~caf | *Rufous Sibia* | Omnivore | r | LC | + | + | + | + |
| 59 | *Hierococcyx sparverioides* | Cuc~spa | *Large Hawk Cuckoo* | Insectivore | s | LC | + | + | + | + |
| 60 | *Hypsipetes leucocephalus* | Hyp~leu | *Black Bulbul* | Omnivore | r | LC | + | + | + | + |
| 61 | *Hypsipetes mcclellandii* | Hyp~mcc | *Mountain Bulbul* | frugivorous | r | LC | + | + | + | + |
| 62 | *Lanius schach* | Lan~sch | *Long-tailed Shrike* | Carnivore | r | LC |  | + |  | + |
| 63 | *Lanius tephronotus* | Lan~tep | *Grey-backed Shrike* | Carnivore | r | LC |  | + |  |  |
| 64 | *Leiothrix lutea* | Lei~lut | *Red-billed Leiothrix* | Omnivore | r | LC, II |  | + |  |  |
| 65 | *Lophura leucomelanos* | Lop~leu | *Kalij Pheasant* | Herbivore | r | LC | + | + |  |  |
| 66 | *Luscinia pectoralis* | Lus~pec | *White-tailed Rubythroat* | Insectivore | r | LC |  |  | + |  |
| 67 | *Megalaima asiatica* | Meg~asi | *Blue-throated Barbet* | frugivorous | r | LC | + | + | + |  |
| 68 | *Megalaima franklinii* | Meg~fra | *Golden-throated Barbet* | frugivorous | r | LC | + | + | + |  |
| 69 | *Megalaima virens* | Meg~vir | *Great Barbet* | frugivorous | r | LC | + | + | + | + |
| 70 | *Megalaima zeylanica* | Meg~zey | *Brown-headed Barbet* | frugivorous | r | LC | + |  |  |  |
| 71 | *Melophus lathami* | Mel~lat | *Crested Bunting* | Omnivore | r | LC |  |  |  | + |
| 72 | *Milvus migrans* | Mil~mig | *Black Kite* | Carnivore | r | LC, II | + | + |  | + |
| 73 | *Minla cyanouroptera* | Min~cya | *Blue-winged Minla* | Insectivore | r | LC |  | + |  |  |
| 74 | *Minla ignotincta* | Min~ign | *Red-tailed Minla* | Insectivore | r | LC |  | + |  |  |
| 75 | *Minla strigula* | Min~str | *Chestnut-tailed Minla* | Insectivore | r | LC |  | + |  |  |
| 76 | *Monticola cinclorhynchus* | Mon~cin | *Blue-capped Rock Thrush* | Insectivore | s | LC | + | + | + | + |
| 77 | *Monticola rufiventris* | Mon~ruf | *Chestnut-bellied Rock Thrush* | Insectivore | r | LC | + |  |  |  |
| 78 | *Monticola solitarius* | Mon~sol | *Blue Rock Thrush* | Insectivore | r | LC | + | + |  |  |
| 79 | *Motacilla cinerea* | Mot~cin | *Grey Wagtail* | Insectivore | r | LC |  |  | + |  |
| 80 | *Muscicapa sibirica* | Mus~sib | *Dark-sided Flycatcher* | Insectivore | s | LC | + | + | + | + |
| 81 | *Myiomela leucura* | Myi~leu | *White-tailed Robin* | Insectivore | r | LC | + | + |  |  |
| 82 | *Myophonus caeruleus* | Myo~cae | *Blue Whistiling Thrush* | Omnivore | r | LC | + | + | + | + |
| 83 | *Nectarinia asiatica* | Nec~asi | *Purple Sunbird* | Nectarivore | r | LC | + |  |  |  |
| 84 | *Niltava macgrigoriae* | Nil~mac | *Small Niltava* | Omnivore | s | LC | + |  |  | + |
| 85 | *Niltava sundara* | Nil~sun | *Rufous-bellied Niltava* | Omnivore | rs | LC |  | + | + |  |
| 86 | *Oriolus traillii* | Ori~tra | *Maroon Oriole* | Omnivore | r | LC | + | + | + |  |
| 87 | *Orthotomus sutorius* | Ort~sut | *Common Tailorbird* | Omnivore | r | LC | + | + |  | + |
| 88 | *Parus major* | Par~maj | *Great Tit* | Insectivore | r | LC | + | + |  | + |
| 89 | *Parus monticolus* | Par~mon | *Green-backed Tit* | Omnivore | r | LC | + | + | + | + |
| 90 | *Parus xanthogenys* | Par~xan | *Black-lored Tit* | Insectivore | r | LC | + | + | + | + |
| 91 | *Passer domesticus* | Pas~dom | *House Sparrow* | frugivorous | r | LC | + | + |  |  |
| 92 | *Passer montanus* | Pas~mon | *Eurasian Tree Sparrow* | Herbivore | r | LC | + | + |  | + |
| 93 | *Pericrocotus ethologus* | Per~eth | *Long-tailed Minivet* | Insectivore | r | LC | + | + | + | + |
| 94 | *Pericrocotus flammeus* | Per~fla | *Scarlet Minivet* | Insectivore | r | LC |  |  |  | + |
| 95 | *Phoenicurus hodgsoni* | Pho~hod | *Hodgson's Redstart* | Omnivore | sw | LC |  |  | + |  |
| 96 | *Phoenicurus ochruros* | Pho~och | *Black Redstart* | Insectivore | m | LC | + |  |  |  |
| 97 | *Phylloscopus chloronotus* | Phy~chl | *Lemon-rumped Warbler* | Insectivore | r | LC |  |  |  | + |
| 98 | *Phylloscopus fuligiventer* | Phy~ful | *Smoky Warbler* | Insectivore | m | LC | + |  |  |  |
| 99 | *Phylloscopus humei* | Phy~hum | *Hume's Warbler* | Insectivore | r | LC |  | + | + | + |
| 100 | *Phylloscopus inornatus* | Phy~ino | *Yellow-browed Warbler* | Insectivore | rm | LC | + |  |  |  |
| 101 | *Phylloscopus maculipennis* | Phy~mac | *Ashy-throated Warbler* | Insectivore | r | LC | + |  |  |  |
| 102 | *Phylloscopus occipitalis* | Phy~occ | *Western Crowned Warbler* | Insectivore | sw | LC |  |  |  | + |
| 103 | *Phylloscopus pulcher* | Phy~pul | *Buff-barred Warbler* | Insectivore | m | LC |  | + |  |  |
| 104 | *Phylloscopus trochiloides* | Phy~tro | *Greenish Warbler* | Insectivore | s | LC | + | + | + | + |
| 105 | *Picus canus* | Pic~can | *Grey-headed Woodpecker* | Insectivore | r | LC | + | + | + |  |
| 106 | *Picus squamatus* | Pic~squ | *Scaly-bellied Woodpecker* | Insectivore | r | LC |  |  | + |  |
| 107 | *Pnoepyga albiventer* | Pno~alb | *Scaly-breasted Wren Babbler* | Omnivore | r | LC |  | + |  |  |
| 108 | *Pnoepyga pusilla* | Pno~pus | *Pygmy Wren Babbler* | Insectivore | r | LC | + |  |  |  |
| 109 | *Pomatorhinus erythrogenys* | Pom~ery | *Rusty-cheeked Scimitar Babbler* | Omnivore | r | LC | + | + | + | + |
| 110 | *Prinia atrogularis* | Pri~atr | *Hill Prinia* | Insectivore | r | LC |  | + | + | + |
| 111 | *Prinia sylvatica* | Pri~syl | *Jungle Prinia* | Insectivore | r | LC |  |  | + | + |
| 112 | *Pycnonotus cafer* | Hyp~caf | *Red-vented Bulbul* | Omnivore | r | LC | + | + | + | + |
| 113 | *Pycnonotus leucogenys* | Hyp~leu | *Himalayan Bulbul* | Omnivore | r | LC | + | + | + | + |
| 114 | *Rhipidura albicollis* | Rhi~alb | *White-throated Fantail* | Insectivore | r | LC | + |  |  |  |
| 115 | *Rhipidura aureola* | Rhi~aur | *White-browed Fantail* | Insectivore | r | LC | + |  |  | + |
| 116 | *Rhyacornis fuliginosus* | Rhy~ful | *Plumbeous Water Redstart* | Omnivore | r | LC |  |  | + |  |
| 117 | *Saxicola caprata* | Sax~cap | *Pied Bushchat* | Insectivore | r | LC |  |  | + | + |
| 118 | *Saxicola ferrea* | Sax~fer | *Grey Bushchat* | Insectivore | r | LC | + | + | + | + |
| 119 | *Saxicola torquata* | Sax~tor | *Common Stonechat* | Insectivore | rw | LC |  |  | + | + |
| 120 | *Seicercus castaniceps* | Sei~cas | *Chestnut-crowned Warbler* | Insectivore | r | LC | + | + |  |  |
| 121 | *Seicercus xanthoschistos* | Sei~xan | *Grey-hooded Warbler* | Insectivore | r | LC | + | + | + | + |
| 122 | *Sitta castanea* | Sit~cas | *Chestnut-bellied Nuthatch* | Insectivore | r | LC | + |  | + |  |
| 123 | *Sitta himalayensis* | Sit~him | *White-tailed Nuthatch* | Omnivore | r | LC | + | + | + |  |
| 124 | *Spilornis cheela* | Spi~che | *Crested Serpent Eagle* | Carnivore | s | LC, II | + |  |  |  |
| 125 | *Stachyris pyrrhops* | Sta~pyr | *Black-chinned Babbler* | Omnivore | r | LC |  | + |  |  |
| 126 | *Stachyris ruficeps* | Sta~ruf | *Rufous-capped Babbler* | Omnivore | r | LC | + |  |  |  |
| 127 | *Streptopelia chinensis* | Str~chi | *Spotted Dove* | Herbivore | r | LC | + | + | + | + |
| 128 | *Streptopelia orientalis* | Str~ori | *Oriental Turtle Dove* | Herbivore | r |  | + | + | + | + |
| 129 | *Surniculus lugubris* | Sur~lug | *Drongo Cuckoo* | Insectivore | s | LC |  |  | + |  |
| 130 | *Tephrodornis gularis* | Tep~gul | *Large Woodshrike* | Insectivore | r | LC |  |  | + |  |
| 131 | *Tesia castaneocoronata* | Tes~cas | *Chestnut-headed Tesia* | Insectivore | r | LC |  |  | + |  |
| 132 | *Treron sphenura* | Tre~sph | *Wedge-tail Green Pigeon* | frugivorous | r | LC | + | + |  | + |
| 133 | *Troglodytes troglodytes* | Tro~tro | *Winter Wren* | Insectivore | r | LC | + |  |  |  |
| 134 | *Turdoides nipalensis* | Tur~nip | *Spiny Babbler* | Insectivore | r | LC, End |  | + |  |  |
| 135 | *Turdus boulboul* | Tur~bou | *Grey-winged Blackbird* | Omnivore | r | LC | + | + | + | + |
| 136 | *Turdus merula* | Tur~mer | *Eurassian Blackbird* | Omnivore | sw | LC | + |  |  | + |
| 137 | *Turdus naumanni* | Tur~nau | *Dusky Thrush* | Omnivore | sw | LC | + |  |  |  |
| 138 | *Turdus obscurus* | Tur~obs | *Eye-browed Thrush* | Omnivore | sw | LC |  |  |  | + |
| 139 | *Turdus ruficollis* | Tur~ruf | *Dark-throated Thrush* | Omnivore | sw | LC |  |  | + | + |
| 140 | *Turdus unicolor* | Tur~uni | *Tickell's Thrush* | Omnivore | s | LC |  |  | + | + |
| 141 | *Urocissa erythrorhyncha* | Uro~ery | *Red-billed Blue Magpie* | frugivorous | r | LC | + | + | + | + |
| 142 | *Yuhina flavicollis* | Yuh~fla | *Whiskered Yuhina* | Insectivore | r | LC | + | + | + |  |
| 143 | *Yuhina gularis* | Yuh~gul | *Striped-throated Yuhina* | Insectivore | r | LC | + | + |  |  |
| 144 | *Zoothera dauma* | Zoo~dau | *Scaly Thrush* | Omnivore | s | LC | + | + |  | + |
| 145 | *Zoothera wardii* | Zoo~war | *Pied Thrush* | Omnivore | s | LC |  |  | + |  |
| 146 | *Zosterops palpebrosus* | Zos~pal | *Oriental White-eye* | Omnivore | r | LC | + | + | + | + |

* r, resident; m, migrant; s, summer visitor (migrant); sw, both summer and winter; rs, both resident and summer visitor; +, bird present.

**Conservation status: LC, IUCN Red list category of least concern; En, Endangered and V, vulnerable and nationally threatened (NT) category; End, endemic; II, Convention on International Trade in Endangered Species (CITES) appendix II.
