# Supplementary figures and images for: Heterogeneous Landscapes on Steep Slopes at Low Altitudes as Hotspots of Bird Diversity in a Hilly Region of Nepal in the Central Himalayas
Source: PLoS One. 2016 Mar 3;11(3):e0150498. doi: 10.1371/journal.pone.0150498 (PMC4777546; doi:10.1371/journal.pone.0150498)

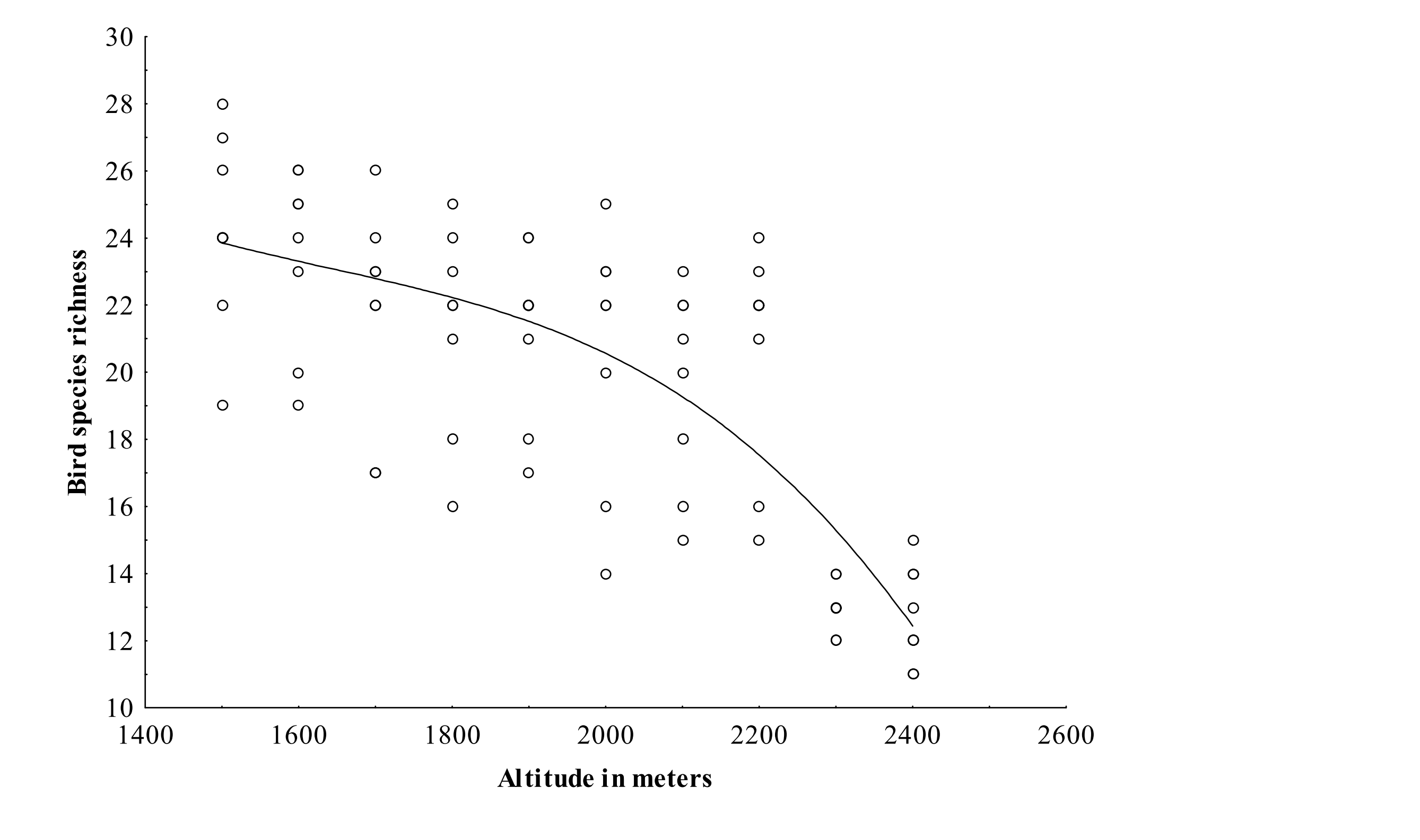

Supplement: S2 Appendix — (TIF) [file pone.0150498.s002.tif]

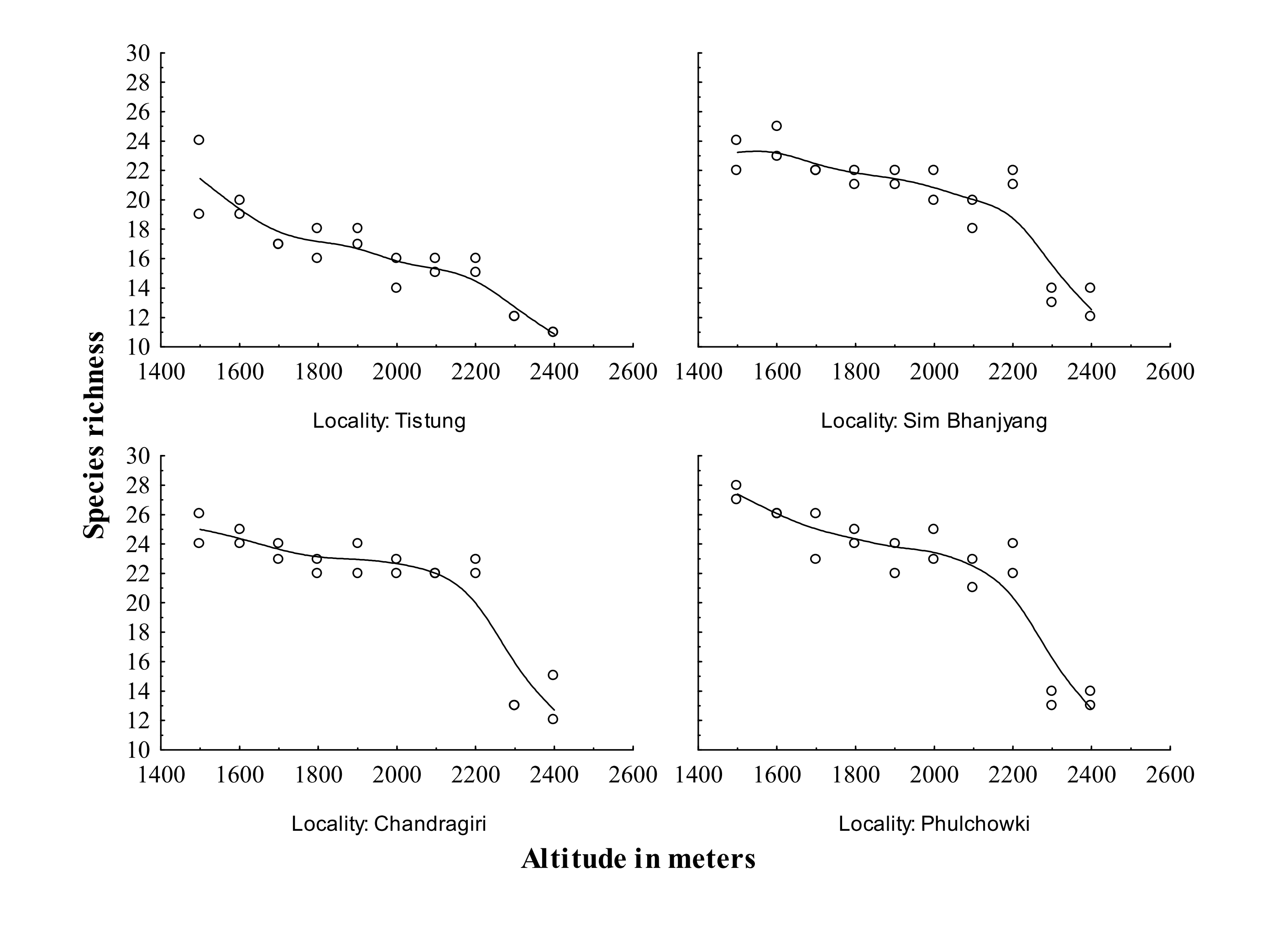

Supplement: S3 Appendix — (TIF) [file pone.0150498.s003.tif]
