# Supplementary material for: Heterogeneous Landscapes on Steep Slopes at Low Altitudes as Hotspots of Bird Diversity in a Hilly Region of Nepal in the Central Himalayas
Source: PLoS One. 2016 Mar 3;11(3):e0150498. doi: 10.1371/journal.pone.0150498 (PMC4777546; doi:10.1371/journal.pone.0150498)
Supplement: S1 Supporting Information — (DOCX) [file pone.0150498.s004.docx]

**Details of Accession Numbers**

All figures are available from the Figshare database. Please see the details of accession number(s) in URL of Web Page Listing the DOI:

**Figures**

Figure 1: https://figshare.com/s/4dd3680be37af4fe270d

Figure 2: https://figshare.com/s/a69b28bcda1974895e9b

Figure 3: <https://figshare.com/s/4db62617310191d4cfdf>

Figure 4: <https://figshare.com/s/eae4e001dc9207fdbbff>

Figure 5: <https://figshare.com/s/7a51ea10bb9458083048>

Figure 6: <https://figshare.com/s/9650af9ed026f3339c9f>

Figure 7: <https://figshare.com/s/422204a116016c52e1a5>

Figure 8: <https://figshare.com/s/260315d758e084b54c29>

**Appendices**

Appendix 2: <https://figshare.com/s/a7de9b6b6fc0bfbbce76>

Appendix 3: <https://figshare.com/s/e21712882d918851d909>

**List of Figures**

List of Figures: <https://figshare.com/s/272f2748042c657316c9>
